# Are Treatment Services Ready for the Use of Big Data Analytics and AI in Managing Opioid Use Disorder?

**DOI:** 10.2196/58723

**Published:** 2025-04-28

**Authors:** Matthew Amer, Rosalind Gittins, Antonio Martinez Millana, Florian Scheibein, Marica Ferri, Babak Tofighi, Frank Sullivan, Margaret Handley, Monty Ghosh, Alexander Baldacchino, Joseph Tay Wee Teck

**Affiliations:** 1 NHS Tayside Ninewells Hospital Dundee United Kingdom; 2 DigitAS Project, Population and Behavioural Science Research Division School of Medicine University of St Andrews St Andrews United Kingdom; 3 Aston Pharmacy School Pharmaceutical & Clinical Pharmacy Research Group College of Health and Life Sciences Aston United Kingdom; 4 Universitat Politècnica de València Valencia Spain; 5 South East Technological University Waterford City Ireland; 6 European Monitoring Centre for Drugs and Drug Addiction Lisbon Portugal; 7 Friends Research Institute Baltimore, MD United States; 8 Department of Epidemiology and Biostatistics University of California San Francisco, CA United States; 9 Department of Medicine Cumming School of Medicine 2500 University Drive NW Calgary, AB Canada

**Keywords:** machine learning, ML, artificial intelligence, AI, algorithm, predictive model, predictive analytics, predictive system, practical model, deep learning, early warning, early detection, big data, opioid use, opioid, opioid use disorder, substance use, substance use disorder

## Abstract

In this viewpoint, we explore the use of big data analytics and artificial intelligence (AI) and discuss important challenges to their ethical, effective, and equitable use within opioid use disorder (OUD) treatment settings. Applying our collective experiences as OUD policy and treatment experts, we discuss 8 key challenges that OUD treatment services must contend with to make the most of these rapidly evolving technologies: data and algorithmic transparency, clinical validation, new practitioner-technology interfaces, capturing data relevant to improving patient care, understanding and responding to algorithmic outputs, obtaining informed patient consent, navigating mistrust, and addressing digital exclusion and bias. Through this paper, we hope to critically engage clinicians and policy makers on important ethical considerations, clinical implications, and implementation challenges involved in big data analytics and AI deployment in OUD treatment settings.

## Introduction

### Opioid Use Disorder and Treatment Policy

Opioid use disorder (OUD) is a global public health crisis and is associated with significant morbidity, mortality, and implications for socioeconomic development [1]. There is a broad range of policy responses to reduce the public health burden of OUD, including demand reduction, supply reduction, harm reduction, and treatment policies [2-4]. Drug demand reduction interventions include public education and communication programs about the risks and harms of opioids and treatment options [2,3]. Supply restriction interventions include reducing unlawful access to opioids through law enforcement and reducing inappropriate lawful access by influencing physician opioid prescribing practices through Prescription Drug Monitoring Programs and clinical guidelines [2,3]. Harm reduction interventions include opioid overdose education and naloxone distribution (OEND) programs, drug checking, syringe service programs, and supervised injection facilities [4]. Treatment policies tend to focus on increasing access to and use of medications for opioid use disorder (MOUD), such as methadone and buprenorphine, and other psychological and behavioral interventions [4].

### Challenges to Policy Implementation

Among the range of policy responses, robust international evidence supports harm reduction interventions and MOUD [2,3,5-7]. Despite this, there is a significant treatment gap across low-, middle-income, and high-income countries [8,9]. In the United States, for example, only 13.4% of people who might have benefitted were able to access MOUD, such as methadone and buprenorphine [10]. Worldwide, only 1 in 12 people in need of treatment for substance use disorder can access it [11]. Furthermore, there is substantial variation across and within countries in the programmatic components, implementation, and quality of the different interventions [9,12-14]. Jin et al [14] illustrate this in their international systematic review of treatment programs offering MOUD, which vary considerably in quality, accessibility, and consistency, limiting its uptake and efficacy among people with OUD.

Further complicating the implementation of policy responses to OUD is the diversity in potential outcomes, not all of which are equally valued. For example, harm reduction interventions intended to reduce the negative impact of drug use without requiring abstinence have been shown to reduce opioid overdose deaths and sequelae of injecting drug use, such as blood-borne virus transmission or soft tissue infections [5-7]. Yet, due to the stigma associated with drug use [15], including by treatment providers [16], there is often poor provision of these interventions in many settings [6]. Many countries emphasize law enforcement approaches to reduce the drug supply over the provision of supervised drug consumption rooms, despite growing evidence of the impact of this intervention in reducing drug overdose events [17]. Similarly, OUD service providers may have different perspectives on how positive outcomes from pharmacotherapy are defined. For some services, a positive outcome is defined as a person becoming abstinent and exiting a treatment program with the assistance of medication. In contrast, for others, it is defined as remaining on MOUD in the long term while reducing harmful illicit use [5,18].

Against this complex and contested environment, there has been widespread support for data-driven systems and surveillance to inform policy, planning, funding, governance, monitoring, and evaluation of OUD interventions and treatment services [19-22]. As with other areas of health care, big data analytics and artificial intelligence (AI) are catalyzing a fundamental shift in what can be accomplished through data-driven systems to tackle the opioid crisis [23]. This includes predictive modeling using large, linked datasets to identify underserved areas with high opioid overdose rates and the development of clinical decision support systems (CDSS) to identify and stratify OUD risk [23-30].

## Defining AI

AI refers to the use of computational systems and mathematical algorithms that simulate human-like intelligence to analyze complex problems [31]. AI is currently a consolidated field of research in health care and is being largely applied in other sectors [32]. The European Commission high-level experts group on AI defines it as “...systems that display intelligent behavior by analyzing their environment and taking actions—with some degree of autonomy—to achieve specific goals” [33]. These types of algorithms, or models, use large volumes of data (namely, big data), which come from heterogeneous sources, to identify patterns, find relationships between data entities, and generate predictions for supporting decision-making.

In the specific context of OUD, AI systems may be able to use a range of data types and sources, including patient records, treatment histories, and demographic information, to generate insights into the characteristics of patients and the consequences that treatments and policies have on the management of related conditions. These types of analyses can be supervised, in which the model knows the outcome, or unsupervised, in which the outcome is unknown and modeling helps to identify groups of patients or outcomes. AI has the potential to enable health care systems to continuously improve their performance by learning from new data. In the context of OUD services, this may include enhancing clinical decision-making with AI-generated predictive insights, personalizing care, and improving operational efficiency.

## Challenges to Deploying AI Applications in Health Care Settings

The large-scale deployment and integration of Al solutions into health care systems are known to have challenges, including the lack of a coherent regulatory framework across jurisdictions [34] and data protection, legal, ethical, and trust issues [35]. However, we argue that there are additional complexities and challenges within the OUD policy and treatment services environment in implementing AI and big data solutions. These include the highly regulated nature of OUD treatment services, the criminalization and stigmatization of people who use drugs, inequities in access to technology and data, and a degree of cognitive dissonance clinicians may have in their dual roles of regulating behavior and providing person-centered care [24,36]. Using our collective experiences as OUD treatment providers, evidence experts, and policy makers, we aim to highlight the challenges to be addressed so that these technologies can be used ethically, effectively, and equitably.

While it is beyond our scope to provide a cutting-edge account of this rapidly evolving field, we begin by providing examples of big data analytics and AI applications in OUD service settings. We then focus on 8 key challenges that OUD treatment services must contend with to make the most of these rapidly evolving technologies.

## Big Data Analytics and AI Applications in OUD Treatment Settings

Big data analytics is characterized by the integration and analysis of a large volume of continuously generated, heterogeneous, and complex data from various sources, including sensors, smartphones, electronic health records (EHRs), results of clinical investigations, and the internet [37]. This contrasts with more traditional research studies with typically fixed boundaries, including clearly defined variables and data collected at specific time points over an established period. Types of data include clinical trial data, anthropometrics (eg, weight and height measurements), demographic information, payer and insurance data, lifestyle, behavioral and psychological traits, continuous physiological measurements, clinical phenotyping (eg, diagnoses, medication use, medical imaging, and procedure results), and process measures captured from mobile and wearable health applications (eg, smartphone apps and text messaging) [37].

**Figure 1:**
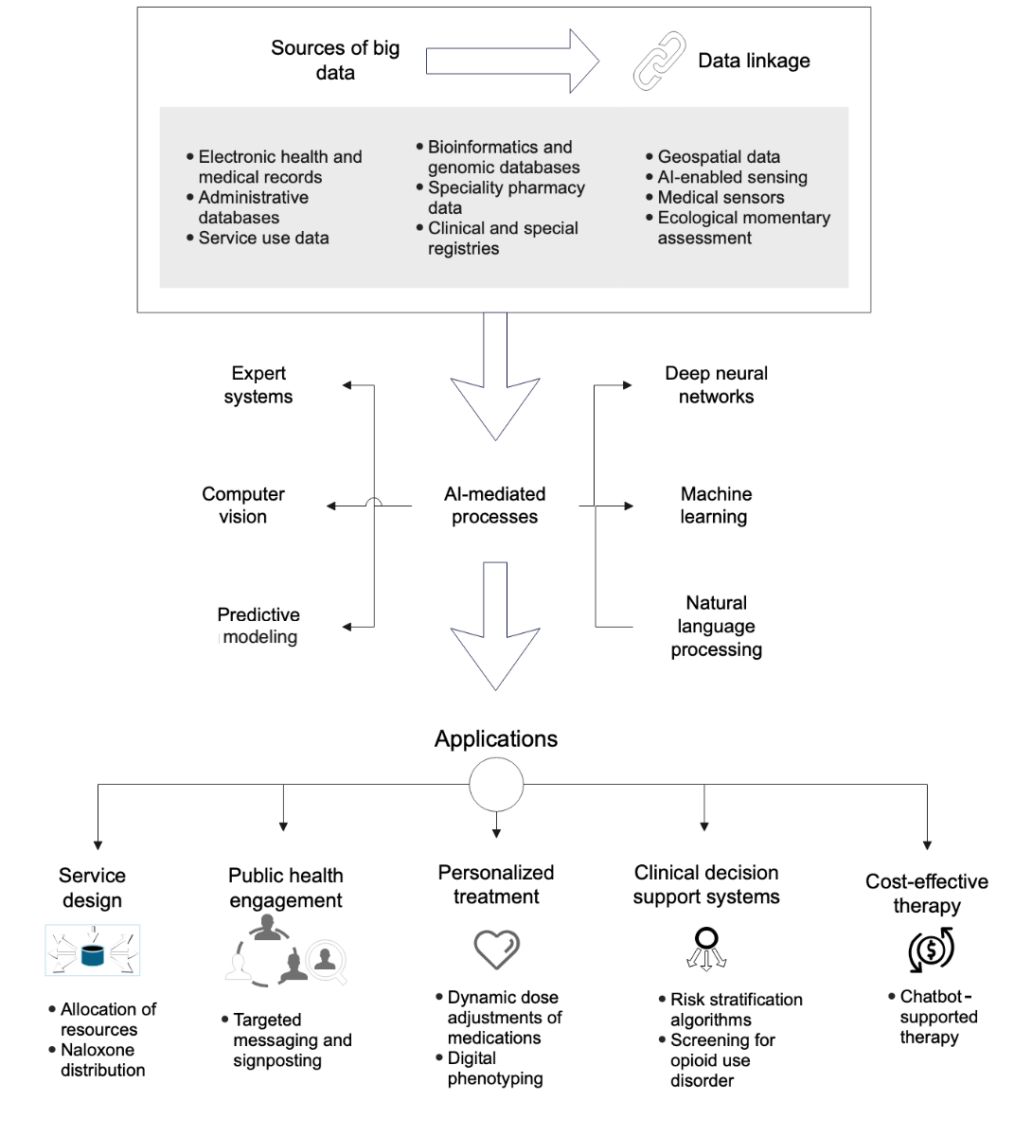
Big data and artificial intelligence (AI) applications for opioid use disorder (OUD) treatment services.

Important technological developments that have expanded the amount of timely and actional health data include continuous digital connectivity through the mobile internet [38] and the digitization of how we interact with each other and our environment [39]. For example, our mobile phones can provide continuous location information and the means to engage in real-time with social media communities [39]. Bluetooth-enabled wearable technology allows the continuous monitoring of our heart rate and rhythm, oxygen levels, sleep patterns, and other physiological measurements [40]. Many health services provide digital interfaces to exchange and store information, creating patient-held EHRs [41]. Figure 1 provides an overview of some AI applications applicable to the OUD treatment setting.

Due to the volume of generated health care data and the velocity at which they are produced, it is beyond the capacity of most health care systems to absorb and respond to every individual data stream [42]. For example, in a system designed to detect and respond to an opioid overdose, physiological information such as respiratory rate will need to be contextualized alongside the individual’s clinical phenotype, and the health care system will need to be primed to respond to a predetermined threshold for action [42]. Advanced statistical methods, the hallmark of AI and big data analytics, have therefore become necessary to extract timely, understandable, and actionable outputs from health care data [43].

Several reviews have described use cases for big data analytics and AI in the management of OUD [23-27,38,44-50]. This includes the discovery of new pharmaceuticals [43], population-level surveillance and public health planning [24,27,48,51,52], OUD or opioid overdose risk prediction [23,24,26,27,44,53-55], prediction models for treatment engagement and retention [23,25,52,54,56,57], generative AI interfaces to provide advice and support [45], and improved monitoring and diagnostics [46,58-60]. Table 1 provides more details on a selection of these applications.

**Table 1:** Practical applications of big data analytics and artificial intelligence (AI) applications relevant to opium use disorder (OUD) treatment services.

Domain, and big data analytics and AI approaches			Examples
**Population-level epidemiology and needs assessment**			
	Contextualizing opioid overdose events with visual representations of neighborhood-built environment conditions [51]	Geospatial data, Google Street View images, nonemergency “311” service requests, and US Census data were used as indicators to produce a high-resolution spatial-temporal analysis, indicating that OUD is influenced by social and neighborhood determinants such as depressing or insecure living environments, poverty, and health issues to inform health policies and guide responses to the opioid crisis.	
	Mapping opioid overdose events against health care access and socioeconomic factors	Spatiotemporal patterns and maps using color to show variation in aggregates of geographical data created from opioid overdose–related emergency department visits, geolocation, data on socioecological factors such as health behaviors, health care, social and economic factors, and physical environment identified that emergency room visit rates were significantly associated with the changes in health care factors (ie, access to care and quality of care) and socioeconomic factors (ie, levels of education, employment, income, family and social support, and community safety) [61].	
	Using social media data to identify trends in opioid use [62-64]	Statistical techniques applied to social media data may provide close to real-time, county-level estimates of overdose mortality, a basis to inform prevention and treatment decisions.	
	Social media analysis and digital phenotyping [65]	Other studies have identified the possibility of detecting a “phenotype” of social media use by text analysis in conditions such as schizophrenia, which may allow new opportunities to support early illness or relapse detection in the community.	
	Using social media to provide targeted interventions for OUD	The social media platform X (formerly known as Twitter) offers a feasible approach to identify some people who use opioids, making it a possible arena to disseminate evidence-based content and facilitate linkage to treatment and harm reduction services [66].	
**Integrated treatment approaches.**			
	Identifying intervention touchpoints for people with OUD across sectors and services	Data linkage studies, for example, between the records of major health and social agencies and the use of machine learning predictive models, have the potential to identify key intervention (touch) points to provide health or social care, treatment for OUD, or harm reduction interventions and can improve understanding of clinical trajectories for people with OUD [67,68].	
	Generative AI and chatbots	Chatbots that offer support, accountability, and some forms of psychotherapy. Preliminary studies show encouraging application in addiction treatment [69-71]	
**Clinical decision-making**			
	Development of clinical decision support algorithms for OUD treatment	Combining evidence-based tools, expert consensus with electronic health care data, and monitoring tools to develop a screening measure, symptom tracking measure, and clinical decision support algorithm necessary to implement measurement-based care for OUD with buprenorphine in primary care [72].	
	Stratifying overdose risk	NarxCare is a proprietary analytic tool that analyses state-mandated prescription databases in the United States to calculate a risk score for possible overdose deaths, which is displayed in the patient’s electronic medical record [28].	
**Tailoring interventions and personalized approaches; personalized medicine or treatment**			
	Predictive analytics to identify high-risk periods for people with OUD and link these with appropriate interventions	Linking ecological momentary assessment data (where participants are prompted by a smartphone app to self-report on various factors such as sleep, stress, pain, craving, and mood), ambulatory physiological assessment using mobile sensors or smartwatches and social media data, and using deep neural networks for predictive analysis may be useful to identify people at high risk of cravings or withdrawal symptoms to receiving dynamic dose adjustments of medications for OUD (eg, methadone and buprenorphine) to improve treatment retention [54].	
**Drug discovery**			
	—^a^	Drug discovery models using generative AI may drive forward potential brain-targeting biological therapeutics for people who use drugs [73].	
**Performance and quality benchmarking**			
	Identifying service level characteristics associated with disengagement from OUD treatment	Predicting premature discontinuation of OUD treatment, using a supervised machine learning approach for analysis of millions of treatment episodes to identify predictors of treatment discontinuation: the most influential risk factors include characteristics of service setting, geographic region, primary source of payment, referral source, employment status, and delays to entering treatment [56].	

^a^Not applicable.

## Implementation Challenges in OUD Treatment Services.

As mentioned earlier, there are important caveats to the potential of big data analytics and AI to transform OUD services, including limited research evidence on the impacts of this technology on treatment outcomes, uncertainties around the regulatory frameworks for the use of AI in health care, and significant translational and implementation obstacles [74-77]. Here, we focus on 8 key challenges that OUD treatment services must contend with to make the most of these rapidly evolving technologies.

### Service Performance Data and Algorithmic Transparency

Given the treatment gap and the lack of evidence-based practice [78], there have been calls to introduce data-driven approaches to monitor and improve OUD treatment service performance [78,79]. For example, the Pew Charitable Trust—a US nonpartisan, data-driven think tank—proposed making intervention impact and quality data publicly available to create accountability at the state level on efforts to tackle the opioid crisis [80]. This proposal advocates for the collection and publication of disaggregated data on the numbers diagnosed with OUD, prescribed MOUD, and supported to remain on treatment for at least 6 months [80]. In 2021, the Scottish Government launched a public health–led MOUD quality standards monitoring program, which incorporated many of the measures recommended by the Pew expert panel [81]. Within 3 years, improvements in treatment access have been observed in Scotland, including a significant shortening in the waiting times to access MOUD [82].

There have been some notable examples of using big data analytics to identify service-level performance gaps [56]. For example, a US-based study used machine learning techniques on a sample of 941,286 OUD treatment episodes collected in 2015-2017 and identified inequities in access to treatment dominated by race, health insurance, and housing status [52]. Another study used granular spatiotemporal data to map area-level access and quality of care against emergency department opioid overdose visits, potentially exposing jurisdictional variability in performance [61]. Nevertheless, barriers to service providers and funders engaging in these approaches may lie in increased public scrutiny, potentially leading to performance management and other sanctions. Furthermore, insights into performance may not be welcome in settings where services are beleaguered by multiple pressures, including underfunding, lack of incentives, competing priorities, and a lack of capacity.

Transparency and accountability are equally important where big data and AI systems are deployed in OUD treatment settings. The term explainable AI describes important aspects of transparency in using predictive algorithms, including its purpose, function, accuracy, and traceability [83]. Furthermore, with regard to function, it is important for clinicians and patients to understand the limits of these predictions, including the data used for training the algorithm and the implications for both accepting and rejecting the output [84]. Unfortunately, the lack of algorithmic transparency is a common finding. In a recent systematic review on the use of AI in mental health research, a lack of transparency was noted in the reporting of methodological flaws relating to statistical processes and data preprocessing [50,85]. This lack of transparency often links back to the proprietary nature of AI algorithms that have been developed as commercial products [85]. However, it may also be an unintentional consequence where black-box deep learning approaches are used or where there is a complexity level that the algorithm creators do not understand [86].

### Clinical Validation

Linked to algorithmic transparency is the clinical validation of AI and big data applications in real-world OUD service settings [74,87]. A recent narrative review on the application of AI in opioid use disorder highlighted the necessity for validation and robust evaluation of these technologies [26]. People in need of OUD treatment are a heterogeneous group with social, psychological, and biological diversity and complexity. This may make them particularly vulnerable to dataset shift, where a machine learning system underperforms as it has been developed from a dataset that is materially different from the one in which it is deployed [88].

The widespread use of unregulated big-data analytics in OUD treatment settings has already been identified. NarxCare (Bamboo Health) is an analytics platform embedded in the EHRs of health care providers in over 45 US states [28]. The platform analyses prescription drug monitoring data and combines them with other health data to provide clinicians with an opioid overdose risk score [28]. Despite no evidence of its safety, effectiveness in reducing overdose deaths, or risk assessment of its potential to increase disparities in access to care, NarxCare is positioned to influence over 1 billion clinical encounters every year in the United States [28]. The pervasiveness of the NarxCare product in everyday patient care underscores the need for more robust regulation of “Software as a Medical Device” products.

It is also important to have a mandated system for monitoring and reporting mistakes and errors arising from using big data and AI analytics tools in health care settings [89]. However, it may be challenging for clinicians to correctly recognize and attribute errors to these tools, considering that algorithm creators themselves do not always understand how their products work. In addition, the lines of accountability for any error detection process have not been defined, for example, whether this would be the manufacturer’s responsibility or an independent regulator [89].

### Navigating the Practitioner-Technology Interface

The interface between AI systems and clinicians is an important yet arguably understudied domain. For example, returning to the use of CDSS for identifying and treating OUD [72] or opioid prescribing [28], it is unclear how to manage the incongruity between an algorithmic and a human clinical decision. What are the medicolegal implications if an algorithmic decision is overridden based on values or ethics-based judgment? Errors in AI and big data outputs are inevitable, yet it remains unclear where responsibility lies if an error output is ignored or acted upon [90]. A clinician may be faced with committing serious harm if, for example, a correct predictor output is ignored or incorrect output is acted on.

In addition, as seen with early technology-clinician interfaces such as radiological screening [91] and medicine interaction prompts [92], there is a risk of “prompt fatigue” with predictive AI models [74]. This is particularly likely where predictive warnings do not seem relevant to typical clinical encounters and may lead to a subconscious dismissal of warnings by clinicians, resulting in error when a correct prediction is given. Indeed, if new technologies become instruments of control and surveillance, thus increasing administrative burden and reducing clinical time and autonomy, clinicians may reject them. The human element of connection has been identified as essential in addiction care by both clinicians and patients [93]. AI and big data interfaces must be designed to be unobtrusive and facilitative of person-centered care to preserve the relational aspect of clinical encounters.

### Capturing the Right Data

Health care data are often siloed into many different systems, such as medical imaging, pathology, EHRs, electronic prescribing tools, and insurance databases. These data become notoriously difficult to link and integrate due to the lack of uniformity in technology infrastructure and privacy and regulatory barriers. The lack of data interoperability limits effective AI model generation, training, and deployment [94]. In addition, the inability of different systems to communicate and share data in real time presents major challenges to the generation of relevant outputs in time-limited situations, for example, in opioid overdose scenarios [95].

Yet, linking diverse data sources has proven to be useful in understanding the clinical trajectories of people with OUD. For example, linked ecological momentary assessment, biosensor, and social media data have been used to increase the predictive accuracy when using AI to model OUD clinical trajectories [54]. However, it is important to note that simply adding more data because they are available does not improve model accuracy. In the work by Marsch et al [54], the data types and streams were carefully selected based on existing evidence, clinical expertise, and judgment.

Furthermore, there are necessary and important regulatory protections to prevent potentially sensitive data from being collected or used outside the context for which they were gathered. This is particularly important in the context of OUD, where prediction models can easily become “surveillance models,” which may impact the individual's privacy and work against their best interests [96]. For example, predictive modeling using social media data may be able to predict future drug use–related behaviors, creating opportunities for intervening and preventing a possible drug overdose [63,64]. However, people using social media have not consented to their data being used this way and may be uncomfortable with it. Their concerns are especially justified in jurisdictions with less robust regulatory and human rights protections, where exposure may lead to arrest or coercive OUD treatment [97].

Data sharing frameworks and models, such as Trusted Research Environment (TRE), have been created in some jurisdictions to address some of the issues raised here [98]. A TRE is a controlled remote access virtual computing domain allowing approved researchers to work with individual-level data but only export aggregate-level results once screened by data custodians [99]. This helps to reduce the costs and risks to organizations when conducting big data analytics studies, which stem from data protection breaches and the operating costs of centralized systems [99,100].

### Responding to and Understanding AI and Big Data Outputs

There is a real risk that services may not keep up with the outputs and insights generated by big data analytics and AI technology. For example, cloud-based AI algorithms can effectively process smartphone-collected data to detect respiratory depression among people using opioids in real time to trigger an automated emergency response [59]. However, this opioid overdose detection and alert system works only if local emergency services have the capacity to respond to this additional data stream. Similarly, modeling studies showing shortfalls in service coverage [51,61] can be helpful if they result in a concerted effort for improvement rather than to generate defensiveness, resentment, or a loss of morale among care providers and funders. While it is laudable that our sector is engaging with new technologies and approaches, it is important that we make sustainable investments in services that have a measurable impact on patient outcomes. In the context of predictive and diagnostic technologies, this must include strengthening existing health care infrastructures to ensure they can respond to an increased demand for their services.

### Obtaining Informed Consent

Health care providers are ethically and legally obliged to obtain informed consent when providing a health intervention [101,102]. Obtaining informed consent is also a key aspect of ethical research practice [103]. This applies also to the use of big data analytics and AI. Andreotta et al [86] have identified 3 issues when obtaining informed consent in relation to the use of big data analytics: the issue of algorithmic transparency (how does it work?), the potential for data to be repurposed (how will it be used?), and providing people with a reasonable alternative should they refuse consent (do I have a choice?).

Unfortunately, in relation to transparency, providing detailed information on the workings of an AI algorithm may not always be helpful to the patient in the decision-making process [86]. Explainable AI, a relatively new research area, attempts to define levels of understanding people may wish to have commensurate with the algorithms’ functions [83]. One of the goals of explainable AI is to standardize how we communicate meaningfully with individuals to empower them to make informed decisions on the risks and benefits of data-sharing [104].

Indeed, the importance of communication about data cannot be understated. Data acquired and processed for a defined health care–related purpose are vulnerable to data security breaches and trafficking, making this a key international regulatory priority [105,106]. The data security landscape is in constant flux, requiring constantly improving systems, including advanced encryption measures [106]. There is, therefore, an ethical obligation when obtaining informed consent to raise awareness of the risks of data security breaches and the measures taken to mitigate against these.

More challenging to incorporate into consenting practices is the issue of repurposed data. AI algorithms may reveal insights and generate secondary uses for the same data that were not anticipated during the original consenting process [107]. While securing informed consent from the original participants of a large dataset is impractical, patients may wish to be protected from data repurposing, which results in actions that are personally detrimental. For example, insights from linked datasets to map intervention touch points for people with OUD [61] can be easily repurposed to highlight areas that become targets of increased policing activity. Robust data protection laws must be in place to reassure patients that sensitive data will not be exploited. Trusted research environments described earlier can act as an arbitrator of public trust by guaranteeing that personal data is not reidentified, providing some reassurances to safeguard against such risks [100].

### Trust in Institutions and Technology

Public trust in institutions and technology is one of the biggest barriers to the growth of big data analytics and AI innovation [108]. Among the most cited trust concerns are safety and security, state overreach, inequalities and bias, and unknown unknowns [108]. Similarly, establishing and maintaining trust is paramount to effective health care delivery [109]. Historically, there have been high levels of public trust in health care institutions, yet this has eroded over the years to the point of crisis in countries such as the United States and the United Kingdom [109].

Unfortunately, trust has long been an issue in OUD treatment settings due to experiences of institutional stigma, discrimination, and conflict [110]. For example, self and social stigma and mistrust of services may result in individuals not disclosing substance use or related social risks such as homelessness or criminal justice involvement [111,112]. Individuals with OUD may also be concerned about their geolocation data being used to penalize them if shared with the police [113]. In other words, people with OUD have had issues of trust relating to safety and security, state overreach, inequalities and bias, and unknown unknowns long before the public in relation to AI. This is justified, as marginalized groups and those whose behaviors are stigmatized or criminalized stand to risk more when trusting their data to public institutions than those in mainstream society [114-116].

One consequence of mistrust is the systematic refusal of a specific group to provide data or consent to data sharing. This results in data absenteeism, excluding them from public health surveillance-based service planning. Data absenteeism is more likely to occur among marginalized or multiply disadvantaged groups, such as people who use drugs, people experiencing homelessness, or those involved with criminal justice [117,118]. The consequence for some groups with OUD is that their needs remain unidentified or planned for at the policy level [117]. There is also evidence to show that some groups miss out on timely data-driven interventions as a result of data absenteeism [119].

Three important considerations need to be addressed when building trust among people with OUD in relation to big data analytics and AI: Do they have a say on the research questions asked using their data? Will the health gains, profits, or benefits from using their data be accessible to them? Will they be disadvantaged by unanticipated socially biased policy decisions based on the data they have provided? The British Colombia Provincial Overdose Cohort is a large linked administrative health dataset on people who have had a drug overdose event [67]. This big data initiative is unique in its active engagement of people with OUD to identify research questions of importance to the affected community, providing responses to the three key trust questions mentioned here [67,120].

Furthermore, policy makers, treatment funders, and providers must take steps to protect people seeking OUD treatment from being disadvantaged should they decline consent to participate in big data initiatives or wish for their data to be withdrawn. The ongoing engagement and consent of people who use drugs, multiple disadvantaged groups, and their advocates is critical to ensuring the impact of data absenteeism is managed and attenuated.

### Digital Exclusion and Bias

People who use drugs and other marginalized and minoritized groups are particularly vulnerable to digital exclusion [121]. Digital exclusion refers to the barriers some face in participating fully in society due to a lack of access or inability to use digital technologies [122]. It is a form of social inequality rooted in the unequal distribution of digital resources. Digital exclusion also contributes to data absenteeism. Rather than declining to share data, the individual has not had the skills, technology, or opportunity to create a digital identity or to generate digitally captured data [122]. Furthermore, these groups are easily ignored in digital co-design and public engagement in data use and AI initiatives [123].

Algorithmic bias in big data analytics describes a systematically repeating predictive or output error that results in prejudicial outcomes against individuals, groups, or social categories due to race, gender, sexuality, socioeconomic grouping, culture, or geographic location [124,125]. AI algorithms are based on existing data that reflect structural inequalities in society, including digital exclusion and prevailing implicit and explicit societal biases against people who use drugs [112]. Consequently, people who use drugs are particularly vulnerable to algorithmic bias [112,126].

The identification of algorithmic bias requires proactive and prospective performance monitoring. An algorithm may, for example, be functional in a general sense in triggering a risk alert for an unwanted clinical outcome yet perform poorly for a specific demographic. Where such discrepancies are identified, more detailed information could be collected for this segment to enable retraining of the model to improve both fairness and accuracy [125]. Similarly, when an algorithm triggers the necessity for clinical contact, the clinician should be provided with guidance on how to interpret the thresholds for action through transparency on the difference between the person’s demographics compared with the algorithmic training cohort [125]. A criticism of the NarxCare overdose risk score is that it has a prominent location within the patient’s EHR, embedding itself into the consultation, yet is not incorporated into the workflow, in that there are no clinically validated score thresholds to trigger an action [28].

### Conclusion

OUD remains a challenging global public health crisis. AI and big data analytics offer new opportunities for data-driven improvements in policy, treatment options, quality, and access. However, there are significant implementation challenges that services need to be prepared for. These technologies expose both patients and services to increased scrutiny and surveillance. We need to ensure that we have made sufficient investments in services to be able to improve and respond to big data and AI outputs. Furthermore, we need to be able to advise our patients, who are already mistrustful of institutions and technology, on the risks and benefits of data sharing. Finally, we need to constantly appraise the limits, validity, legitimacy, and potential for bias in using AI algorithms in our practice. Our clinical expertise and critical scientific skills are needed more than ever to balance against the tempting automaticity of AI and big data analytics. The 8 challenges identified here must be addressed if these new technologies are to be used ethically, effectively, and equitably.

## Abbreviations

AI: artificial intelligence
CDSS: clinical decision support systems
EHR: electronic health record
MOUD: medications for opioid use disorder
OEND: opioid overdose education and naloxone distribution
OUD: opioid use disorder
TRE: Trusted Research Environments
